# Cerebroprotective Effects of the TLR4-Binding DNA Aptamer ApTOLL in a Rat Model of Ischemic Stroke and Thrombectomy Recanalization

**DOI:** 10.3390/pharmaceutics16060741

**Published:** 2024-05-30

**Authors:** Alicia Aliena-Valero, Macarena Hernández-Jiménez, Mikahela A. López-Morales, Eva Tamayo-Torres, María Castelló-Ruiz, David Piñeiro, Marc Ribó, Juan B. Salom

**Affiliations:** 1 Unidad Mixta de Investigación Cerebrovascular, Instituto de Investigación Sanitaria La Fe, 46026 Valencia, Spain; alicia_aliena@iislafe.es (A.A.-V.); mikahela_lopez@iislafe.es (M.A.L.-M.); maria.castello@uv.es (M.C.-R.); 2 AptaTargets S.L., 28035 Madrid, Spain; d.pineiro@aptatargets.com (D.P.); marcriboj@aptatargets.com (M.R.); 3 Departamento de Farmacología y Toxicología, Facultad de Medicina, Universidad Complutense de Madrid, 28040 Madrid, Spain; 4 Departamento de Fisioterapia, Universidad de Valencia, 46010 Valencia, Spain; 5 Departamento de Fisiología, Universidad de Valencia, 46010 Valencia, Spain; eva.tamayo@uv.es; 6 Departamento de Biología Celular, Biología Funcional y Antropología Física, Universidad de Valencia, 46100 Valencia, Spain; 7 Unidad de Ictus, Departamento de Neurología, Hospital Vall d’Hebron, 08035 Barcelona, Spain

**Keywords:** aptamers, ApTOLL, cerebroprotection, immunomodulation, inflammation, ischemic stroke, Toll-like receptor 4

## Abstract

ApTOLL, a TLR4 modulator aptamer, has demonstrated cerebroprotective effects in a permanent ischemic stroke mouse model, as well as safety and efficacy in early phase clinical trials. We carried out reverse translation research according to STAIR recommendations to further characterize the effects and mechanisms of ApTOLL after transient ischemic stroke in rats and to better inform the design of pivotal clinical trials. Adult male rats subjected to transient middle cerebral artery occlusion were treated either with ApTOLL or the vehicle intravenously at different doses and time-points. ApTOLL was compared with TAK-242 (a TLR4 inhibitor). Female rats were also studied. After neurofunctional evaluation, brains were removed for infarct/edema volume, hemorrhagic transformation, and histologic determinations. Peripheral leukocyte populations were assessed via flow cytometry. ApTOLL showed U-shaped dose-dependent cerebroprotective effects. The maximum effective dose (0.45 mg/kg) was cerebroprotective when given both before reperfusion and up to 12 h after reperfusion and reduced the hemorrhagic risk. Similar effects occurred in female rats. Both research and clinical ApTOLL batches induced slightly superior cerebroprotection when compared with TAK-242. Finally, ApTOLL modulated circulating leukocyte levels, reached the brain ischemic tissue to bind resident and infiltrated cell types, and reduced the neutrophil density. These results show the cerebroprotective effects of ApTOLL in ischemic stroke by reducing the infarct/edema volume, neurofunctional impairment, and hemorrhagic risk, as well as the peripheral and local immune response. They provide information about ApTOLL dose effects and its therapeutic time window and target population, as well as its mode of action, which should be considered in the design of pivotal clinical trials.

## 1. Introduction

In the reperfusion era, recanalization through thrombolysis and/or mechanical thrombectomy leads to an improvement in clinical outcomes in approximately 30% of acute ischemic stroke (AIS) patients [1]. However, only approximately 15% of stroke patients are eligible for reperfusion therapies and, among those with a successful recanalization, 70% still suffer from moderate–severe disabilities or die. Therefore, there is a clear and urgent medical need to develop new medicinal products for AIS, targeting therapeutic pathways other than reperfusion [2,3]. In this context, ApTOLL has recently emerged as a real therapeutic option for cerebroprotection in stroke.

ApTOLL (aptamer 4FT) is a single-strain DNA aptamer able to reduce inflammation in stroke models [4] via the modulation of the Toll-like receptor type 4 (TLR4), a key mediator of the brain inflammatory response induced by several insults, including cerebral ischemia [5,6,7,8]. To date, the preclinical pharmacodynamic effects of ApTOLL administration have been confirmed in different animal disease models (brain and myocardial ischemia) and species (mice, rats and pigs) [4,9,10], but definite evidence for transient ischemic stroke is lacking.

With this background, our group started the clinical development of ApTOLL with the objective of testing ApTOLL in patients who have suffered an AIS. A first-in-human phase I clinical trial (ApTOLL-FIH-01; NCT04742062) was initially conducted, demonstrating ApTOLL’s safety in healthy subjects [11]. Next, ApTOLL clinical development focused on AIS patients in a phase Ib/IIa clinical trial (APRIL Study; NCT04734548) [12]. APRIL’s results confirmed ApTOLL’s safety and efficacy in AIS patients with large vessel occlusion eligible for endovascular therapy (EVT). For the first time in the context of a cerebroprotective drug for AIS, ApTOLL was associated with a clinical effect of reducing the mortality, final infarct volume, functionality impairments at 72 h, and disability at 90 days as compared to a placebo [13].

These promising clinical findings await confirmation from larger pivotal trials. Therefore, we are continuing to carry out reverse translation research to extend preclinical evidence and better inform upper phase clinical trials to achieve definite conclusions. In the present study, we addressed updated STAIR XI recommendations for preclinical studies in stroke therapies [14]. We assessed ApTOLL’s cerebroprotective effects in an independent laboratory, both in male and ovariectomized female Wistar rats subjected to closely monitored intraluminal filament transient middle cerebral artery occlusion (tMCAO), the model of choice to determine the preclinical efficacy of drugs as adjuncts to EVT [15]. This study aimed to define ApTOLL’s dose–response characteristics and time window, with both histological and functional outcomes for brain damage. The efficacy of ApTOLL from a clinical-grade batch was also assessed. The effects of the TLR4 antagonist TAK-242 (Resatorvid) were assessed for comparison. Finally, to gain insight into ApTOLL’s mechanisms of action, we studied the modulation of inflammatory cell populations in peripheral blood, as well as the presence of ApTOLL in the brain tissue and the effect on the local immune response.

## 2. Materials and Methods

### 2.1. Animals

**Figure 1:**
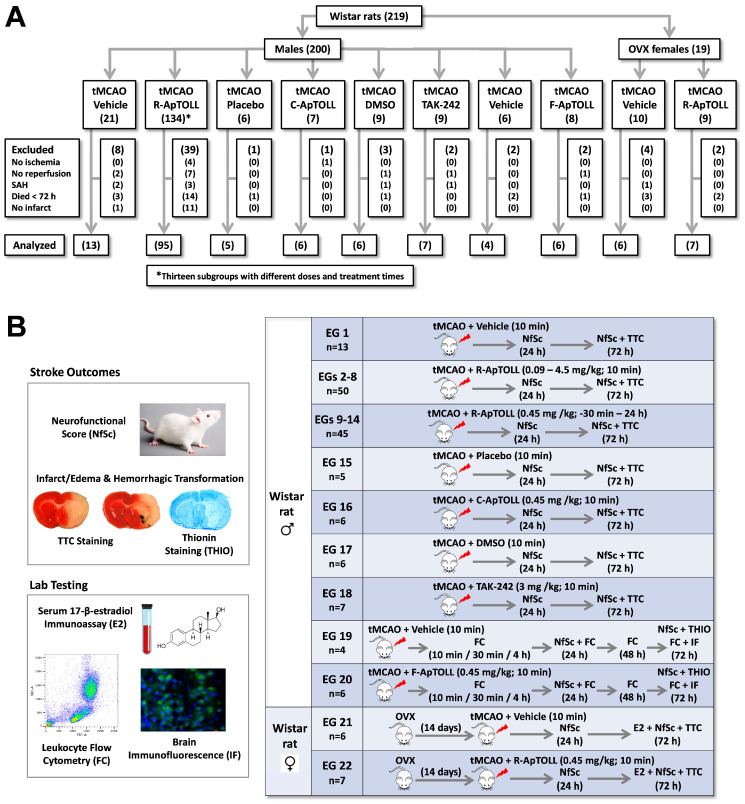
CONSORT-style flow diagram and experimental schedule. (**A**) Progress of all rats included in the study. Rats were excluded from quantitative assessment of outcomes according to the following predefined criteria: (1) cortical perfusion (CP) did not drop after filament insertion (no ischemia); (2) CP did not recover at all after filament withdrawal (no reperfusion); (3) subarachnoid hemorrhage (SAH) during filament insertion/withdrawal; (4) death before 72 h; and (5) no TTC-detectable brain infarction in spite of a right ischemia–reperfusion pattern. OVX: ovariectomized; tMCAO: transient middle cerebral artery occlusion; R-ApTOLL, research-grade ApTOLL; C-ApTOLL, clinical-grade ApTOLL; F-ApTOLL, Alexa Fluor 488-conjugated ApTOLL. (**B**) Time course of the experimental schedule for each of the twenty groups of Wistar male rats (EGs1-20) and the two groups of female rats (EGs21-22), including procedures (OVX and tMCAO), treatments (vehicle, ApTOLL, and TAK-242), methods for stroke outcome assessment (NfSc: neurofunctional score; TTC: 2,3,5-triphenyl tetrazolium chloride staining; and THIO: thionin staining), and lab tests (E2: 17-β-estradiol immunoassay; FC: flow cytometry, and IF: immunofluorescence). The number of analyzed rats (n) is indicated.

A total of 200 male (300–350 g) and 19 female (275–325 g) 12-week-old Wistar rats from Charles River (Barcelona, Spain) were used in this study (Figure 1A). They were housed under standard conditions, including ad libitum feeding and drinking and a 12 h light/12 h dark cycle (the light was turned on at 8:00 h and turned off at 20:00 h). This study was designed and conducted according to the STAIR/RIGOR guidelines [14,16] regarding physiological monitoring, gender consideration, power analysis and sample size calculations, simple randomization, predefined exclusion criteria, allocation concealment, blinded assessment of several outcomes at different endpoints, and conflict of interest statements. The ARRIVE (Animal Research: Reporting of In Vivo Experiments) guidelines were followed (https://arriveguidelines.org/; accessed on 21 April 2024).

### 2.2. Ovariectomy

Two weeks before inducing an ischemic stroke, female rats were anesthetized (face mask, 3.5% sevoflurane in 80% medicinal air plus 20% O_2_) and subjected to a bilateral ovariectomy. Buprenorphine (subcutaneous [s.c.], 0.05 mg/kg) was used to provide analgesia. Serum 17-β-estradiol (E2) was measured in samples obtained during euthanization, by means of the IMMULITE 1000 Estradiol immunoassay kit (Siemens Healthcare Diagnostics Products, Getafe, Madrid, Spain) according to the manufacturer’s instructions.

### 2.3. Ischemic Stroke: Transient Focal Cerebral Ischemia

The animal model (tMCAO) was always studied from 9:00 h to 11:00 h, that is, during the resting phase of the circadian cycle in rats. Animals were anesthetized by intraperitoneal (i.p.) injection of 5 mg/kg diazepam, 100 mg/kg ketamine and 0.3 mg/kg atropine. Inhalatory anesthesia was maintained with 0.5–1% sevoflurane in 80% medicinal air plus 20% O_2_. Right tMCAO was performed by following the intraluminal nylon filament procedure as originally described [17] and adapted to our experimental setup [18]. Before the surgery, the filament was dipped in heparin to prevent blood clotting and hyperthermia associated with hypothalamic lesions [19]. The procedure included continuous monitoring of cerebrocortical laser-Doppler flow (cortical perfusion, CP), arterial blood pressure and core temperature and discontinuous measurements of blood glucose at pre-ischemia (basal), ischemia and reperfusion stages. For mechanical recanalization, the intraluminal filament was withdrawn after 60 min of MCAO and reperfusion was monitored for 30 min. Buprenorphine (s.c., 0.05 mg/kg) was used to provide analgesia. The animals were subjected to neurofunctional evaluation 24 h and 72 h after the ischemic insult and euthanized by intracardiac injection of KCl (200 mg/kg) or perfused under anesthesia to obtain the brain according to specific requirements for each determination. Whole blood and serum samples were also obtained.

### 2.4. Experimental Groups, Treatments and Exclusion Criteria

Male animals were randomly assigned to appropriated control (PBS + 1 mM MgCl_2_, clinical placebo or DMSO, according to the experimental group), ApTOLL, Alexa Fluor 488-conjugated ApTOLL, or TAK-242 treatments. Research-grade ApTOLL or Alexa Fluor 488-conjugated ApTOLL (Aptus Biotech, Madrid, Spain), structured and lyophilized from a PBS + 1 mM MgCl_2_ solution, was dissolved in sterile water. Clinical-grade ApTOLL (Reig Jofre, Barcelona, Spain) was dissolved in sterile water. TAK-242 (Resatorvid, MedChemExpress, South Brunswick, NY, USA) was dissolved in DMSO. For the dose–response study, ApTOLL was intravenously (i.v.) injected 10 min after reperfusion at one of seven different doses (0.09, 0.225, 0.45, 0.675, 0.9, 2.25, and 4.5 mg/kg). To determine the therapeutic time window, ApTOLL (0.45 mg/kg) was i.v. injected at one of seven different time points with respect to reperfusion onset (−30 min, +10 min, +2 h, +6 h, +9 h, +12 h, and +24 h). Control animals received a vehicle. For sex consideration, female animals were randomly assigned to vehicle or ApTOLL treatments (0.45 mg/kg, i.v. injected 10 min after reperfusion). To establish the biological effect of the ApTOLL clinical batch to be administered in AIS patients enrolled in the APRIL trial, 0.45 mg/kg was i.v. injected 10 min after reperfusion in male animals. Control animals received a clinical placebo. For the TLR4 antagonist comparison, TAK-242 (3 mg/kg) was i.p. injected 10 min after reperfusion in male animals. TAK-242 control animals received solvent (1 mL/kg DMSO).

A total of 58 (29.00%) male and 6 (31.58%) female rats were excluded from quantitative assessment of outcomes according to the predefined criteria (Figure 1A). Allocation was concealed from the investigator carrying out the surgical procedure, including drug administration, and applying the exclusion criteria. Male and female animals analyzed in the 22 experimental groups of vehicle- or drug-treated rats are detailed in the experimental schedule (Figure 1B).

### 2.5. Assessment of Outcomes

The primary outcome measure was brain infarction, and secondary outcome measures were brain edema, hemorrhagic transformation (HT), and neurofunctional status, which were assessed by an investigator blinded to treatment allocation.

Neurological function was measured as reported in [18], with slight modifications. The severity of functional deficits was scored by assessing (a) spontaneous activity (moving/exploring = 0, no moving or moving only when pushed = 1); (b) circling to the left (none = 0, circling when elevated by the tail and pushed = 1, circling without displacement, spinning = 2); (c) parachute reflex: protective abduction of forelimbs (symmetrical = 0, asymmetrical, contralateral forelimb retracted = 1); and (d) resistance to left forepaw stretching (not allowed = 0, allowed = 1). The total score could range from 0 (no neurological deficits) to 5 (highest neurological deficits) or 6 (death).

The brain infarct volume was determined ex vivo using the 2,3,5-triphenyltetrazolium chloride (TTC) vital staining method [20], followed by morphometric analysis [18]. Briefly, rats were euthanized under anesthesia and the brain was sliced in seven 2 mm thick coronal sections, which were immersed in a 2% solution of TTC in saline solution at 37 °C for 10 min, fixed in 10% phosphate-buffered formalin (pH 7.4) overnight, and digitally photographed for image analysis. The infarct area in the slices and total infarct volume were calculated with edema correction separately for cortical and subcortical regions. The difference between raw and corrected infarcted hemisphere is equal to the edema volume. Occurrence of gross HT was also macroscopically assessed in the infarct [21].

### 2.6. Flow Cytometry in Peripheral Blood

Peripheral blood was serially collected at different times post-treatment (10 min, 30 min, 4 h, 24 h, 48 h, and 72 h) in EDTA tubes, which were incubated with ammonium chloride potassium (ACK) lysis buffer for 10 min at room temperature (#00-4300-54, eBioscience™ 10X RBC Lysis Buffer, Invitrogen, Thermo Fisher Scientific, Waltham, MA, USA). Afterwards, blood was centrifuged at 400× *g* for 5 min at 4 °C. The supernatant was removed, and the pellet was resuspended in FACS buffer. We used Zombie UV™ fixable live/dead cells (#423107, Biolegend, San Diego, CA, USA) for exclusion of dead cells. Staining for leukocyte populations was carried out with antibodies against CD45-PerCP-Vio700 ([1:10], #130-126-112, Miltenyi, Cologne, Germany), CD45R-PE ([1:10], #130-106-774, Miltenyi), CD3-PEVio770 ([1:10], #130-103-773, Miltenyi), CD8-BV711 ([1:100], #740724, BD Biosciences, Franklin Lakes, NJ, USA), CD4-VioBlue ([1:50], #130-123-286, Miltenyi), CD11b/c-APC-Vio770 ([1:50], #130-121-130, Miltenyi), CD161-APC ([1:10], #130-102-713, Miltenyi), and Granulocytes-BV602 ([1:100], #743055, BD Biosciences). After incubating for 10 min in the dark, cells were washed with PBS and fixed with Cytofix (#554655, BD Biosciences). Data acquisition was performed in an SLRFortessa cytometer (BD Biosciencess) with FACS Diva software (BD Biosciences; https://www.bdbiosciences.com/en-us/products/software/instrument-software/bd-facsdiva-software; accessed on 21 April 2024). Data were analyzed with FlowJo™ v10.6.0 software (FlowJo LLC, BD Biosciences).

### 2.7. Fluorescent Immunohistochemistry in the Brain

Three days following tMCAO, animals were anesthetized with 5 mg/kg diazepam and 100 mg/kg ketamine and perfused with cold saline for 10 min followed by 20 min of 4% paraformaldehyde (PFA, #P6148, Sigma-Aldrich, Madrid, Spain). Thereafter, brains were left for 24 h in 4% PFA, washed 3 × 5 min with cold 1X PBS and then cryoprotected in 30% sucrose for three days. Cryoprotected brains were flash-frozen on dry ice, mounted in Tissue-Tek OCT compound (Sakura Finetek, Torrance, CA, USA), and cut using a Leica CM 1950 cryostat (Leica Biosystems, Nussloch, Germany) to obtain 20 μm thick coronal sections located from 0.2 to −1.8 mm from the bregma, which were stored at −20 °C.

For immunofluorescence, sections were blocked with 10% normal goat serum (#S-1000-20, Vector Laboratories, Newark, CA, USA) in PBS with 0.3 or 0.8% Triton X-100 for 1 h at room temperature and then incubated overnight at 4 °C with the following antibodies: anti-NeuN ([1:250], mAb #12943, Cell Signaling, Danvers, MA, USA), anti-GFAP ([1:500], #840001, Biolegend, San Diego, CA, USA), anti-Iba1 ([1:500], #019-19741, Fujifilm Wako Pure Chemical Corporation, Chuo-ku, Osaka, Japan) and anti-neutrophils ([1:2000], #S-C348181, LifeSpan BioSciences, Shirley, MA, USA). Afterwards, sections were incubated with Alexa Fluor 568 goat anti-rabbit IgG secondary antibody ([1:200], #A-11036 Invitrogen, Waltham, MA, USA) for 1 h at room temperature, followed by nuclei staining with DAPI (#A1001, ITW Reagents, Milan, Italy) and mounted with FluorSave (#345789, Millipore, Darmstadt, Germany). Adjacent sections were stained with thionine to delineate the infarct area. Immuno-positive cells were visualized using a Leica DM 2500 fluorescence microscope (Leica Biosystems, Nussloch, Germany), and images were taken using 20× and 40× objectives.

### 2.8. Statistical Analysis

The sample size was estimated for the primary outcome infarct size on the basis of previous results [4]. Accepting an alpha risk of 0.05 and a beta risk of 0.2 in a one-sided test, 9 subjects were necessary in each group to recognize a statistically significant minimum difference of 6 units between any pair of groups assuming that 8 groups exist. The common deviation was assumed to be 2.8. A drop-out rate of 30% was anticipated.

The results of quantitative continuous variables were expressed as means ± SEM. Categorical ordinal neurofunctional scores were expressed as medians (Q1, Q3). Data analysis was performed using GraphPad Prism version 9.00 (GraphPad Software Inc., San Diego, CA, USA). Statistical comparisons were made between groups using Fisher’s exact test (for exclusion and HT rates), a Student’s *t*-test (for serum E2 levels and neutrophil counts), one-way ANOVA followed by Dunnett’s multiple comparisons post hoc test or two-way ANOVA followed by Sidak’s multiple comparisons post hoc test (for infarct and edema volumes and for peripheral leukocyte counts) and a Kruskal–Wallis test followed by Dunn’s multiple comparisons post hoc test (for neurofunctional scores). Differences were considered significant at *p* < 0.05.

## 3. Results

Body weight values were similar in all the experimental groups, except for the expected difference between age-matched male and female rats. Arterial blood pressure, serum glucose and core temperature were in the physiologic range and similar in all the experimental groups. No intra-ischemic hyperthermia episodes (≥39 °C) were recorded. The reduction in cerebral perfusion during ischemia was comparable in all the groups, as was the extent of perfusion increases during reperfusion. Similar proportions of animals were excluded in control groups (18 out of 52, 34.62%) and treated groups (46 out of 167, 27.54%; *p* = 0.383).

### 3.1. Cerebroprotective Effects of ApTOLL: Dose–Response and Therapeutic Time Window

**Figure 2:**
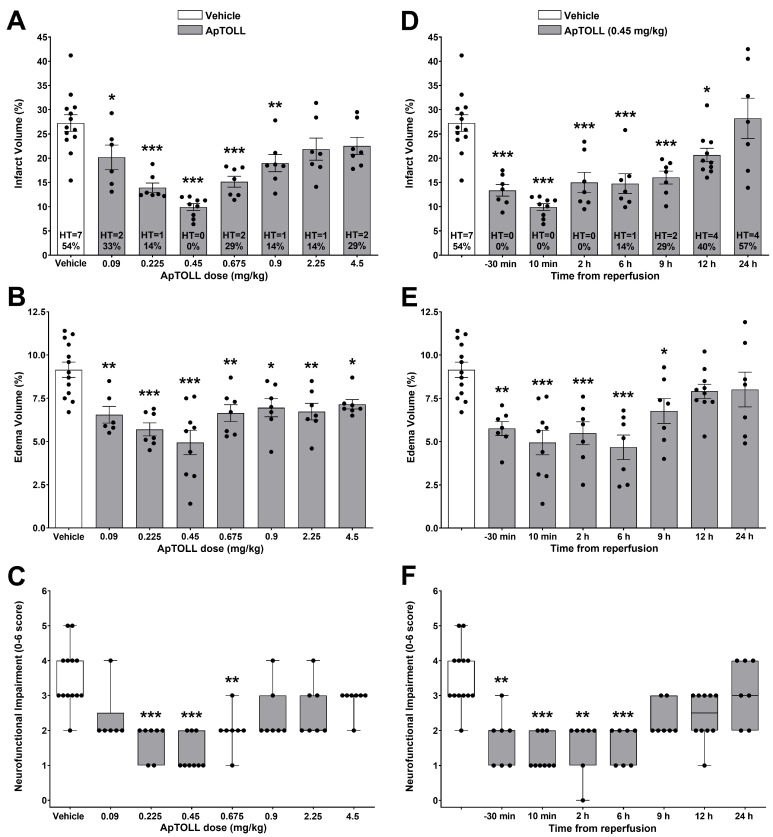
Cerebroprotective effects of ApTOLL in male Wistar rats subjected to transient middle cerebral artery occlusion (tMCAO). (**A**–**C**) Dose-dependent effects of ApTOLL (0.09, 0.225, 0.45, 0.675, 0.9, 2.25, and 4.5 mg/kg), i.v. 10 min post-tMCAO, on infarction, including the absolute and relative occurrence of hemorrhagic transformation (HT), (**A**) and edema volumes (**B**), as well as on neurofunctional impairment (**C**), at 72 h after tMCAO. (**D**–**F**) Therapeutic time window for the effects of ApTOLL (0.45 mg/kg), i.v. injected before (−30 min) or after (+10 min, +2 h, +6 h, +9 h, +12 h, and +24 h) tMCAO, on infarction, including the absolute and relative HT occurrence, (**D**) and edema volumes (**E**), as well as on neurofunctional impairment (**F**), at 72 h after tMCAO. Data are means ± SEM (**A**,**B**,**D**,**E**) and median [Q1, Q3] (**C**,**F**). Statistical analysis: one-way ANOVA followed by Dunnett’s multiple comparisons post hoc test (**A**,**B**,**D**,**E**) and Kruskal–Wallis test followed by Dunn’s multiple comparisons post hoc test (**C**,**F**). Significantly different from vehicle (* *p* < 0.05, ** *p* < 0.01, and *** *p* < 0.001).

A wide range of ApTOLL doses (0.09, 0.225, 0.45, 0.675, 0.9, 2.25, and 4.5 mg/kg), 10 min post tMCAO, were used to assess the effects on stroke-induced infarct and edema volumes, as well as on neurofunctional impairment, in male animals. Figure 2A–C show U-shaped dose-dependent significant cerebroprotective effects 72 h after tMCAO (*n* = 6–13; *p* < 0.05), with maximal reductions in brain damage (Figure 2A,B) and neurofunctional impairment (Figure 2C), as well as no HT occurring at the dose of 0.45 mg/kg. All doses considered, the occurrence of HT was significantly lower in ApTOLL- than in vehicle-treated animals (19.05% versus 53.85%, *p* < 0.05, *RR* = 0.56 [95% CI 0.28–0.89]; Figure 2A). While 17.65% of vehicle-treated animals died before 72 h, 12.50% died after ApTOLL treatment (*p* = 0.691). Also, 5.88% of vehicle- and 9.38% of ApTOLL-treated animals (*p* > 0.99) showed no TTC-detectable infarction.

Injection of ApTOLL (0.45 mg/kg) induced cerebroprotective effects with a wide therapeutic time window. As shown in Figure 2D–F, both intra-ischemic (30 min before reperfusion) and post-reperfusion injections were significantly cerebroprotective (*n* = 7–13; *p* < 0.05). Beneficial effects 72 h after tMCAO were obtained in terms of a significant infarct volume reduction with ApTOLL injected up to 12 h, no HT occurring up to 2 h (Figure 2D), a significant edema reduction up to 9 h (Figure 2E), and a significant neurofunctional improvement up to 6 h post reperfusion (Figure 2F). All times considered, the occurrence of HT was significantly lower in ApTOLL- than in vehicle-treated animals (20.00% versus 53.85%, *p* < 0.05, *RR* = 0.58 [95% CI 0.29–0.92]; Figure 2D). While 17.65% of vehicle-treated animals died before 72 h, 10.29% died after ApTOLL treatment (*p* = 0.411). Also, 5.88% of vehicle- and 10.29% of ApTOLL-treated animals (*p* > 0.99) showed no TTC-detectable infarction.

### 3.2. Sex-Dependent Cerebroprotective Effects of ApTOLL

**Figure 3:**
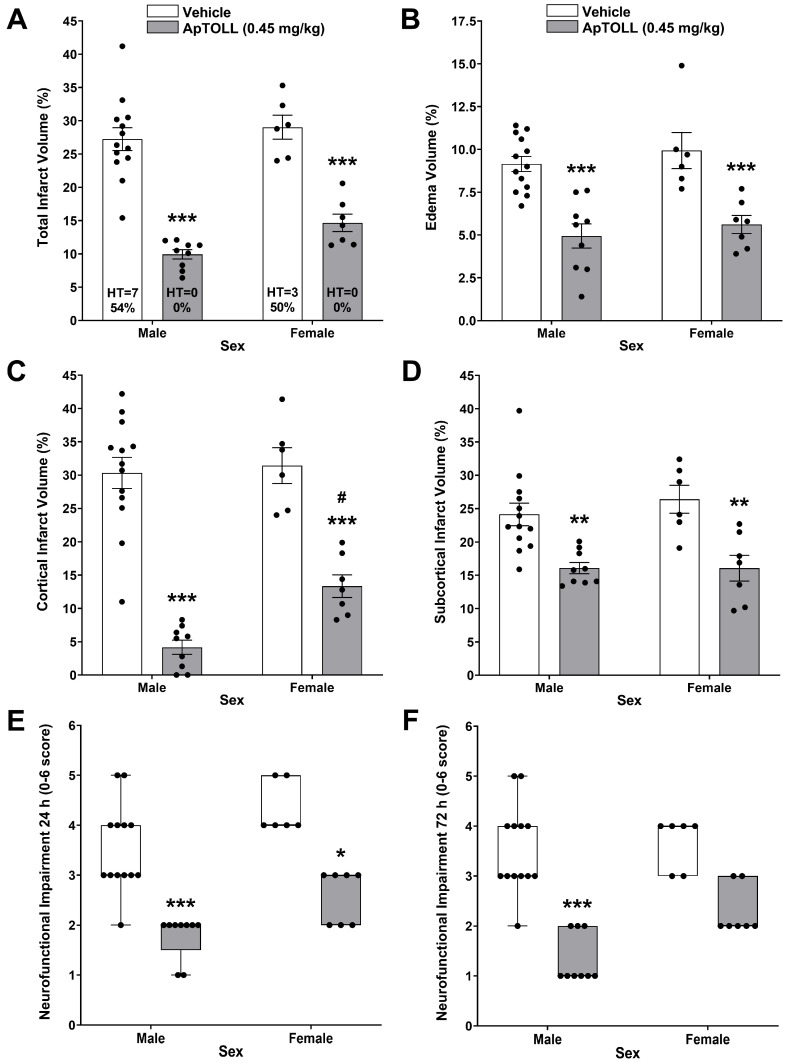
Sex-dependent cerebroprotective effects of ApTOLL in Wistar rats subjected to transient middle cerebral artery occlusion (tMCAO). Comparative effects in male and female rats of ApTOLL (0.45 mg/kg), i.v. 10 min post tMCAO, on total infarction, including the absolute and relative occurrence of hemorrhagic transformation (HT), (**A**) and edema volumes (**B**); on cortical (**C**) and subcortical (**D**) infarct volumes, at 72 h after tMCAO; and on neurofunctional impairment at 24 h (**E**) and 72 h (**F**) after tMCAO. Data are means ± SEM (**A**–**D**) and medians [Q1, Q3] (**E**,**F**). Statistical analysis: two-way ANOVA followed by Sidak’s multiple comparisons post hoc test (**A**–**D**) and a Kruskal–Wallis test followed by Dunn’s multiple comparisons post hoc test (**E**,**F**). Significantly different from vehicle (* *p* < 0.05, ** *p* < 0.01, and *** *p* < 0.001). Significantly different from ApTOLL in males (^#^ *p* < 0.05).

The cerebroprotective effects of ApTOLL (0.45 mg/kg), 10 min post tMCAO, were compared in male and ovariectomized female animals. Both vehicle- and ApTOLL-treated female animals showed reduced serum E2 levels (10.20 ± 1.40 pg/mL, *n* = 6 versus 13.26 ± 0.69 pg/mL, *n* = 7, *p* = 0.06). Similar significant reductions in total infarct and edema volumes were induced in male (*n* = 9–13; *p* < 0.001) and female (*n* = 6–7; *p* < 0.001) animals, and no HT occurred in ApTOLL-treated male and female animals (Figure 3A,B). A regional assessment of infarction showed that, although ApTOLL significantly reduced the cortical infarct volume in male (*n* = 9–13; *p* < 0.001) and female (*n* = 6–7; *p* < 0.001) animals, the reduction was significantly lower in female animals (*p* < 0.05). However, the subcortical infarct volume was significantly reduced to the same extent (*p* < 0.01) in both sexes (Figure 3C,D). Neurofunctional improvement induced by ApTOLL at 24 h was less significant in female (*p* < 0.05) than in male (*p* < 0.001) animals, and was absent in female (*p* = 0.08) animals at 72 h (Figure 3E,F).

### 3.3. Cerebroprotective Effects of Clinical-Grade ApTOLL and the Small-Molecule-Specific TLR4 Inhibitor TAK-242

**Figure 4:**
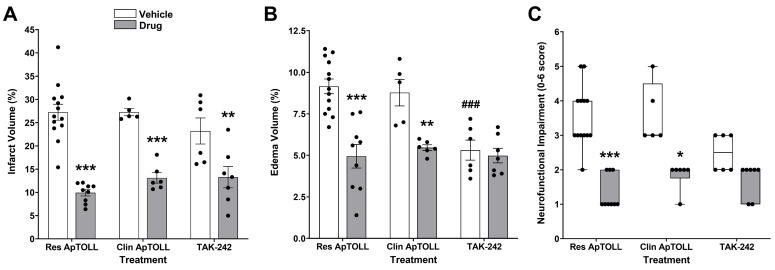
Cerebroprotective effects of clinical-grade ApTOLL and the TLR4 inhibitor TAK-242 in male Wistar rats subjected to transient middle cerebral artery occlusion (tMCAO). Comparative effects of research-grade ApTOLL (Res ApTOLL, 0.45 mg/kg; i.v.), clinical-grade ApTOLL (Clin ApTOLL, 0.45 mg/kg; i.v.), and TAK-242 (3 mg/kg; i.p.), 10 min post-tMCAO, on infarct (**A**) and edema volumes (**B**), as well as on neurofunctional impairment (**C**), at 72 h after tMCAO. Data are means ± SEM (**A**,**B**) and medians [Q1, Q3] (**C**). Statistical analysis: two-way ANOVA followed by Sidak’s multiple comparisons post hoc test (**A**,**B**) and a Kruskal–Wallis test followed by Dunn’s multiple comparisons post hoc test (**C**). Significantly different from vehicle (* *p* < 0.05, ** *p* < 0.01, and *** *p* < 0.001). Significantly different from Res ApTOLL vehicle (^###^ *p* < 0.001).

The efficacy of clinical-grade ApTOLL/placebo was assessed (0.45 mg/kg, 10 min post-tMCAO) in male animals. Placebo-treated animals showed similar results to those obtained in research vehicle-treated animals. When compared to the placebo, clinical-grade ApTOLL-treated animals showed significant brain damage reductions (*n* = 5–6; *p* < 0.01) and neurofunctional improvements (*p* < 0.05), similar to the beneficial effects observed with the research-grade ApTOLL batch (Figure 4A–C).

The effects of TLR4 antagonist TAK-242 (3 mg/kg, i.p. 10 min post-tMCAO) were assessed for comparison. The TAK-242 vehicle (DMSO) showed a slight reduction in brain damage and neurofunctional impairment, which was statistically significant for edema reduction, when compared to the ApTOLL vehicle (*n* = 6–13; *p* < 0.001). When compared to DMSO treatment, TAK-242-treated animals showed a significantly lower infarct volume (*n* = 6–7; *p* < 0.01), but not significantly different edema volumes (*p* = 0.98) or neurofunctional impairment (*p* = 0.63) (Figure 4A–C).

### 3.4. Effects of ApTOLL on Circulating Leukocyte Populations in Peripheral Blood

**Figure 5:**
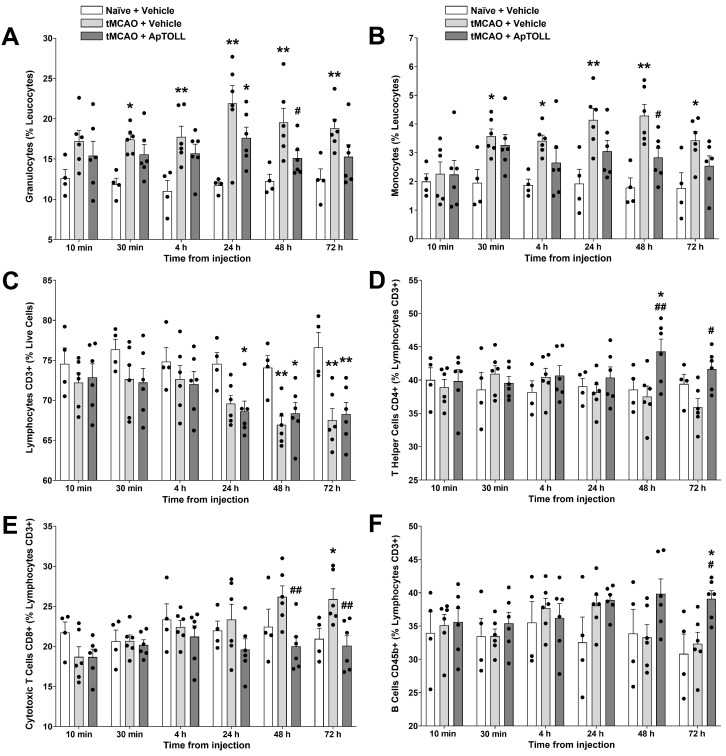
Effects of ApTOLL on leukocyte populations in peripheral blood of male Wistar rats subjected to transient middle cerebral artery occlusion (tMCAO). Effects of ApTOLL (0.45 mg/kg), i.v. 10 min post tMCAO, on peripheral blood levels of granulocytes (**A**), monocytes (**B**), total lymphocytes (**C**), T helper cells (**D**), cytotoxic T cells (**E**), and B cells (**F**), at 10 min, 30 min, 4 h, 24 h, 48 h, and 72 h post injection. Data are means ± SEM. Statistical analysis: two-way ANOVA followed by Sidak’s multiple comparisons post hoc test. Significantly different from naïve + vehicle (* *p* < 0.05, ** *p* < 0.01). Significantly different from tMCAO + vehicle (^#^ *p* < 0.05, ^##^ *p* < 0.01).

Blood samples from naïve-vehicle-treated, tMCAO-vehicle-treated, or tMCAO-ApTOLL-treated animals (0.45 mg/kg, 10 min post-tMCAO) were obtained at 10 min, 30 min, 4 h, 24 h, 48 h, and 72 h post injection and analyzed by flow cytometry (FC). Granulocyte and monocyte levels rapidly increased after tMCAO when compared to naïve animals, being significantly higher from 30 min to 72 h and peaking at 24–48 h (*n* = 4–6; *p* < 0.01). In contrast, ApTOLL-treated animals did not show significant differences to naïve animals, except for increased granulocyte levels at 24 h (*p* < 0.05). In fact, ApTOLL-treated animals showed significantly lower granulocyte and monocyte levels than vehicle-treated animals at 48 h post tMCAO (*p* < 0.05) (Figure 5A,B). Total CD3+ lymphocyte levels were significantly decreased both in vehicle- and ApTOLL-treated animals at 24–72 h post tMCAO when compared with naïve animals (*n* = 4–6; *p* < 0.05; Figure 5C). Regarding lymphocyte subpopulations, CD4+ T Helper cell levels were not modified by tMCAO when compared to naïve animals. However, ApTOLL-treated animals showed a significant increase at 48 h when compared to vehicle-treated animals (*n* = 4–6; *p* < 0.01), which was maintained at 72 h post tMCAO (*p* < 0.05; Figure 5D). CD8+ cytotoxic T cells increased at 48 h and became significantly different from naïve animals at 72 h post tMCAO. At these time points, ApTOLL-treated animals showed significantly reduced cytotoxic T cell levels when compared to vehicle-treated animals (*n* = 4–6; *p* < 0.01; Figure 5E). Additionally, CD45b+ B cells were significantly increased in ApTOLL-treated animals when compared to vehicle-treated animals at 72 h post tMCAO (*n* = 4–6; *p* < 0.05; Figure 5F). Finally, no significant differences were detected in natural killer cell levels among the three experimental groups. 

### 3.5. ApTOLL in the Brain: Cell Types

**Figure 6:**
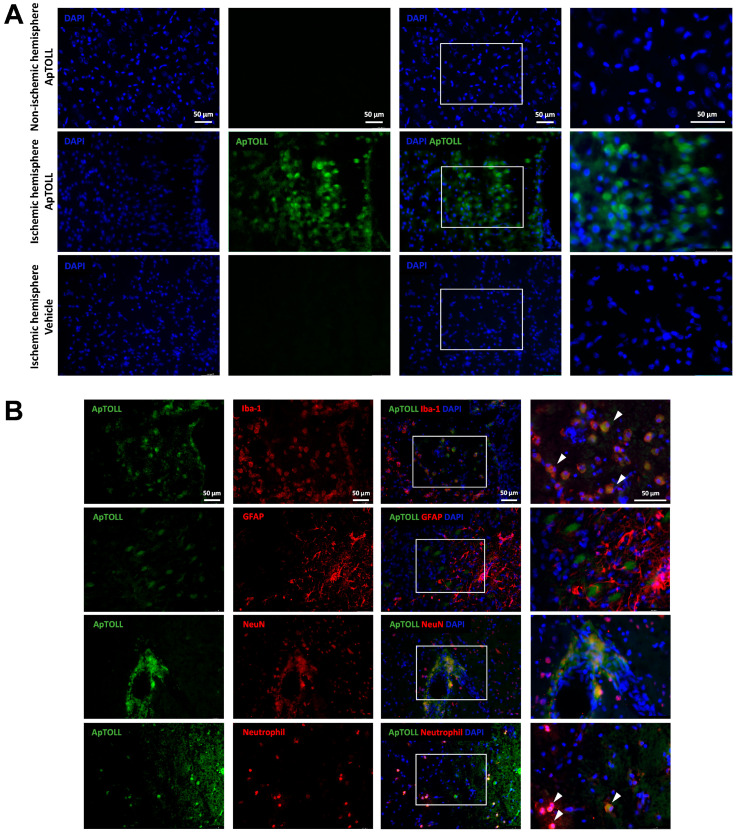
Presence of Alexa Fluor 488-conjugated ApTOLL in the brain of male Wistar rats subjected to transient middle cerebral artery occlusion (tMCAO). (**A**) Representative fluorescence images of brains of animals treated with Alexa Fluor 488-conjugated ApTOLL (0.45 mg/kg) via i.v. 10 min post tMCAO (ischemic hemisphere, **upper** panel; non-ischemic hemisphere, **middle** panel) or vehicle (ischemic hemisphere, **lower** panel) at 72 h after tMCAO. (**B**) Representative immunofluorescence images of brains (ischemic hemisphere) of animals treated with Alexa Fluor 488-conjugated ApTOLL (0.45 mg/kg, i.v. 10 min post tMCAO) at 72 h after tMCAO. From **top** to **bottom**, panels show ApTOLL signals together with immunolabeling for Iba-1 (microglia), GAFP (astroglia), NeuN (neurons), and neutrophils, respectively. Nuclei were counterstained with 4′,6-diamidino-2-phenylindole (DAPI). White arrows in the higher magnification images (right side) point to the colocalization of microglia and neutrophils with ApTOLL.

Brain slices of animals treated with Alexa Fluor 488-conjugated ApTOLL (0.45 mg/kg) or vehicle, 10 min post tMCAO, were obtained at 72 h and observed via microscopy immunofluorescence. Alexa Fluor 488-conjugated ApTOLL was present in the ischemic hemisphere, but not in the non-ischemic hemisphere of ApTOLL-treated animals, nor in the ischemic hemisphere of vehicle-treated animals (Figure 6A).

Alexa Fluor 488-conjugated ApTOLL colocalized with activated Iba-1-positive microglia cells showing ameboid-like morphology and with infiltrated neutrophils in the ischemic hemisphere, but not with GFAP-positive astroglia cells or NeuN-positive neuronal bodies (Figure 6B).

**Figure 7:**
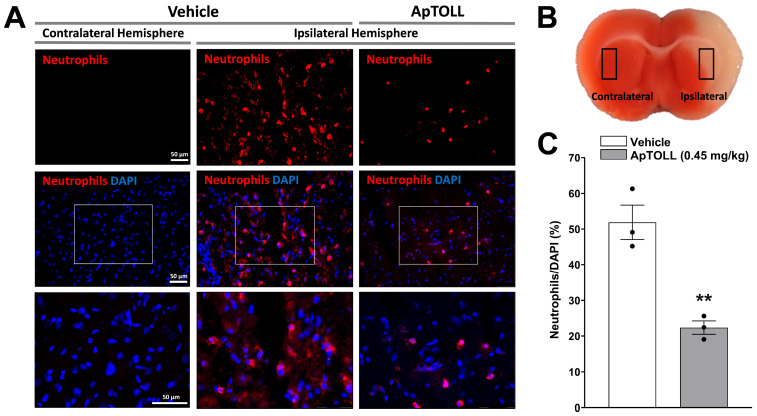
Effect of ApTOLL on neutrophil infiltration in the brain of male Wistar rats subjected to transient middle cerebral artery occlusion (tMCAO). (**A**) Representative immunofluorescence images of the brain ischemic (ipsilateral) hemisphere of animals treated with ApTOLL (0.45 mg/kg) i.v. 10 min post tMCAO or the vehicle at 72 h after tMCAO and of the non-ischemic (contralateral) hemisphere treated with the vehicle, with immunolabeling for neutrophils. Nuclei were counterstained with 4′,6-diamidino-2-phenylindole (DAPI). (**B**) Representative TTC-stained brain slice showing the localization of immunofluorescence images. TTC: 2,3,5-triphenyl tetrazolium chloride. (**C**) Quantitative effect of ApTOLL on the density of infiltrated neutrophils in the ischemic hemisphere. Data are means ± SEM. Statistical analysis: Student’s *t*-test. Significantly different from vehicle (** *p* < 0.01).

Moreover, the ischemic hemisphere of ApTOLL-treated animals showed a significantly lower density of infiltrated neutrophils at 72 h post tMCAO compared with vehicle-treated animals (*n* = 3; *p* < 0.01). In contrast, neutrophils were not observed in the vehicle-treated, non-ischemic hemisphere (Figure 7).

## 4. Discussion

In recent years, ApTOLL, an anti-inflammatory drug with proven safety and cytoprotective effects, both at the preclinical [4,9,10] and clinical levels [11,12,13], has emerged as having real promise for cerebroprotection after stroke. The results from early phase clinical studies strongly support the conduction of large clinical trials to demonstrate ApTOLL’s efficacy in a huge population of AIS patients. To design these trials properly, there is a need to collect more preclinical information on transient cerebral ischemia. Thus, we conducted, for the first time, the whole characterization of ApTOLL in a suitable model of tMCAO [15] according to the STAIR recommendations [14].

First, we performed a dose–response study to assess ApTOLL’s effects on ischemic/reperfusion brain damage up to 72 h after the insult. In line with previous results [4], ApTOLL given at 0.45 mg/kg reduced the infarct volume after tMCAO, but it was also effective at a wide range of doses (0.09–0.9 mg/kg). Interestingly, our results show a U-shaped dose–response curve in the ability of ApTOLL to reduce brain damage and neurofunctional impairment. U-shaped dose–response curves (i.e., hormesis) have been widely documented, with the underlying biological mechanisms likely related to overcompensation responses [22]. Therefore, U-shaped responses raise important issues for toxicological risk assessment and in the establishment of clinical endpoints [23]. Interestingly, ApTOLL also reduced edema at all the doses used in a 50-fold range (0.09–4.5 mg/kg). Cerebral edema is a serious complication associated with ischemia/reperfusion injuries and inflammatory reactions in ischemic stroke and an important risk factor for adverse outcomes [24]. Therefore, both infarct and edema volume reductions could contribute to the neurofunctional improvement observed in the present study. These results suggest the need to evaluate multiple outcomes, as well as to carefully design future ApTOLL dose-finding clinical trials in stroke patients.

Regarding the therapeutic time window for ApTOLL administration, our results demonstrate the cerebroprotective effects of ApTOLL given either before reperfusion or up to 12 h after reperfusion, far beyond the 6 h previously reported in permanent middle cerebral artery occlusion (pMCAO) in mice [4]. Circadian rhythms may affect the response to therapy in stroke [25]. Since our therapeutic time window included treatments in both the resting and active phases in rodents, a certain modulation of the protective effect of ApTOLL by the circadian cycle cannot be ruled out and deserves further research. Taking into account the differences in metabolism, anatomy and physiology between rats and humans, extending the window of ApTOLL’s cerebroprotection to 12 h in rats could mean the possibility of administering ApTOLL in humans up to 72 h after stroke onset [26,27]. Importantly, this extended ApTOLL therapeutic time window could also imply cerebroprotective options for those patients included in the late time window of EVT (up to 24 h) [28,29] and even longer.

HT is a common complication in AIS, which worsens the stroke outcome and increases mortality. Factors associated with an increased risk of HT include reperfusion therapy, with inflammation and the immune system as important contributors [30]. Our results show a reduced risk of HT after ApTOLL treatment and prove ApTOLL’s beneficial effect in a mechanical thrombectomy model. Of note, we also found promising trends demonstrating that ApTOLL treatment reduces the early mortality risk within 3 days from stroke onset and increases the chances of the stroke resulting in non TTC-detectable infarcts.

Biologic sex and sociocultural gender both contribute to differences in stroke risk factors, assessment, treatment, and outcomes [31]. The molecular mechanisms leading to ischemic cell death differ in the two sexes, and these effects may be independent of circulating hormone levels [32]. Therefore, best practices for improving the translatability of findings for strokes encourage the use of both sexes in preclinical studies. ApTOLL’s cerebroprotective effects both in male and female animals were also tested. Since naturally fluctuating estrogen levels in female animals during the estrous cycle have been linked to the extent of stroke brain damage [33], ovariectomized rats were used to avoid a potential confounding effect of estrogen. Ovariectomy causes a so-called surgically induced menopause, leading to a deficiency of circulating estrogens [34]. In our study, serum estrogen levels were similar in ApTOLL- and vehicle-treated female rats, and expectedly below the level reported in metestrus rats [35]. Of note, at the same ApTOLL dose (0.45 mg/kg), the reduction in the cortical infarct volume was smaller and the neurofunctional improvement was less durable in females when compared to male animals. Sex-related differences in immune functions could be an important factor in stroke, as infarcted tissue in the brain produced a stronger inflammatory response in female rats, consistent with increased immune responses [36]. Whether this could underlie sex-related differences in the results of therapeutic intervention with ApTOLL deserves further research. Ischemic stroke is relatively uncommon among premenopausal women, but the risk increases with advancing age. Stroke rates roughly double among postmenopausal women at 55–64 years of age. Estradiol levels decrease from 7- to 10-fold between pre- and post-menopause [37]. Therefore, using a model of stroke in menopausal female rats is clinically relevant.

In an effort to ensure the translation of our results to the clinical situation, we conducted studies with both ApTOLL and placebo clinical batches, as well as used a TLR4 antagonist (TAK-242) already undergoing clinical trials for inflammation-associated conditions [38]. First, we confirmed the absence of effect of the clinical placebo in our experimental conditions, as it was previously demonstrated for the research-grade vehicle batch. Importantly, the cerebroprotective effects of clinical-grade ApTOLL mirrored the benefits obtained with research-grade ApTOLL, confirming the biological activity of this batch at the preclinical level. We also compared the effects of ApTOLL with those of TAK-242, which has previously been used to reduce inflammation in rodent pMCAO [39] and tMCAO models [40,41]. The TAK-242 solvent (DMSO) has established protective effects against brain edema produced by cryogenic lesions combined with a metabolic blocker in albino rabbits [42] or tMCAO in rhesus monkeys [43]. Of note, our results showed that the solvent accounted in part for the cerebroprotective effects of TAK-242, particularly in terms of edema reductions, in line with previous studies showing cerebroprotection by DMSO after both pMCAO and tMCAO in rats [44,45]. In contrast, the vehicle did not contribute to the cerebroprotective effects of ApTOLL, which were slightly superior to those of TAK-242, under the same experimental conditions.

To gain insight into ApTOLL’s mechanisms of action, we assessed the effects of ApTOLL on leukocyte populations in peripheral blood. Our results show that ApTOLL modifies the stroke-induced inflammatory response at the peripheral level from 24 to 72 h after reperfusion. Circadian biology may also influence inflammation and immune responses after a stroke [25,46]. Since the tMCAO procedure and blood collection in vehicle- and ApTOLL-treated animals were always conducted within the resting phase of the circadian cycle in rodents, no effect of circadian rhythms was expected to modulate the effects of ApTOLL on leukocyte counts. In our experimental conditions, the ischemia/reperfusion process induced early increases in granulocytes and monocytes and a late increase in cytotoxic T lymphocytes. These increases were prevented by the administration of ApTOLL, thus supporting its peripheral anti-inflammatory effect after stroke. In addition, ApTOLL was able to increase the levels of T helper and B cell populations at later time points (48 and 72 h). The increase in T helper cells could be due to an increase in regulatory T cells (Tregs), an immunosuppressive subset of peripheral T helper cells crucial for maintaining immune homeostasis [47]. In fact, the protective effects of T cells after ischemic stroke are mainly exerted by circulating Treg cells [48]. On the other hand, an enhancement in regulatory B cells might have a protective role, due largely to their ability to secrete anti-inflammatory IL-10 and to up-regulate and recruit IL-10-secreting Treg cells [49]. Our results are in line with those obtained in mice showing leukocyte counts as surrogate markers of peripheral inflammation after stem cell treatment [50]. Along with already utilized clinical, neurophysiological, and neuroimaging biomarkers, a blood-derived multi-biomarker panel is emerging as a reasonable approach to enhance the predictive power of stroke prognostic models [51]. Therefore, the results obtained in this study could be translated to human peripheral immune responses after an AIS by using flow cytometry analysis as a surrogate blood biomarker of the anti-inflammatory ApTOLL action in clinical trials.

At the brain tissue level, ApTOLL was present in the ischemic hemisphere, but not in the non-ischemic hemisphere, suggesting that ApTOLL is able to cross the damaged blood–brain barrier and reach the ischemia–reperfusion lesion, in line with previous results in pMCAO mice [4]. Moreover, ApTOLL colocalized with activated Iba-1-positive microglia cells showing an ameboid-like morphology and with infiltrated neutrophils in the ischemic hemisphere. Therefore, ApTOLL is able to bind and potentially modulate the function of resident and infiltrating immune cells in the ischemic brain [52]. Our results show a huge infiltration of neutrophils in the ischemic hemisphere, but not in the contralateral side, in agreement with the overwhelming neutrophil infiltration previously reported in rat [53] and mouse [54] tMCAO models. ApTOLL also reduced the density of infiltrated neutrophils in the ischemic hemisphere. Whether ApTOLL could have other effects on the dynamics of cerebral immune cell accumulation [54] and/or specifically on TLR4-mediated neutrophil programming and function after stroke [55,56] deserves further research.

Due to the lack of successful cerebroprotective drugs in clinical trials so far, here, we aimed to enhance the current knowledge regarding the effect of ApTOLL in preclinical settings to support the design of further clinical trials. Therefore, the goals were (1) to establish the best doses and therapeutic time window of administration, (2) to study the potential differences in the response between male and female subjects, (3) to determine the efficacy of the clinical batch which is expected to be used in patients, and (4) to further characterize the ApTOLL’s in vivo mechanism of action of regulating inflammation, as well as to compare its effects with an established TLR4 inhibitor (TAK-242). We did not study the effects of ApTOLL in old or comorbid animals, nor the long-term effects beyond 3 days, because of the already published results obtained in the APRIL clinical trial where ApTOLL showed promising effects at 90 days in a pooled population of AIS patients, in which the mean age was 70 years and subjects with hypertension, hyperlipidemia and diabetes were included [13].

## 5. Conclusions

Our results show the cerebroprotective effects of ApTOLL in ischemic stroke by reducing the infarct/edema volume, neurofunctional impairment, and hemorrhagic risk, as well as peripheral and local immune responses. ApTOLL’s effects provide information about its dose response, therapeutic time window, target population, sex differences, and mechanism of action. Our preclinical results have important implications for ApTOLL clinical trials in the pipeline. The cerebroprotective effects of intra-ischemically administered ApTOLL support the design of new clinical trials to assess the protective effect of ApTOLL even before starting reperfusion treatment (i.e., in ambulances). Also, there is room for allometric scaling to convert higher cerebroprotective ApTOLL doses from rodents to humans, as well as for considering a therapeutic time window far beyond 6 h. Potential sex differences in ApTOLL’s effects and inflammation-related biomarkers could also be considered in the design and analysis of larger ApTOLL clinical trials regarding personalized medicine.

## Data Availability

The datasets used and/or analyzed during the current study to support the study findings are available from the corresponding authors on reasonable request. The data are not publicly available because are part of an ongoing study.
